# Supplementary material for: Chemically induced mutations in a MutaMouse reporter gene inform mechanisms underlying human cancer mutational signatures
Source: Commun Biol. 2020 Aug 14;3:438. doi: 10.1038/s42003-020-01174-y (PMC7429849; doi:10.1038/s42003-020-01174-y)
Supplement: Supplementary file 1 — Supplementary Information [file 42003_2020_1174_MOESM1_ESM.pdf]

SUPPLEMENTARY FIGURES

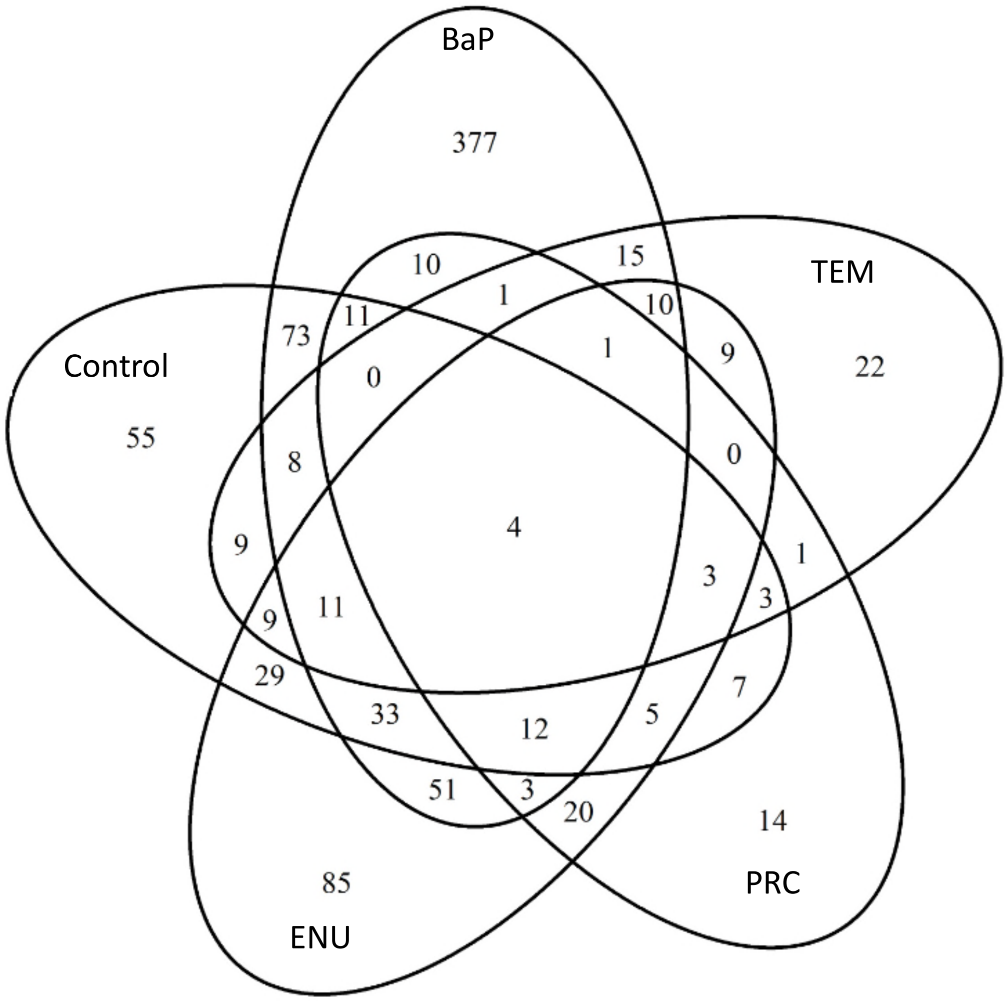

Supplementary Figure 1. Venn diagram showing the overlap of unique mutations in the controls and four chemical treatment groups**.**

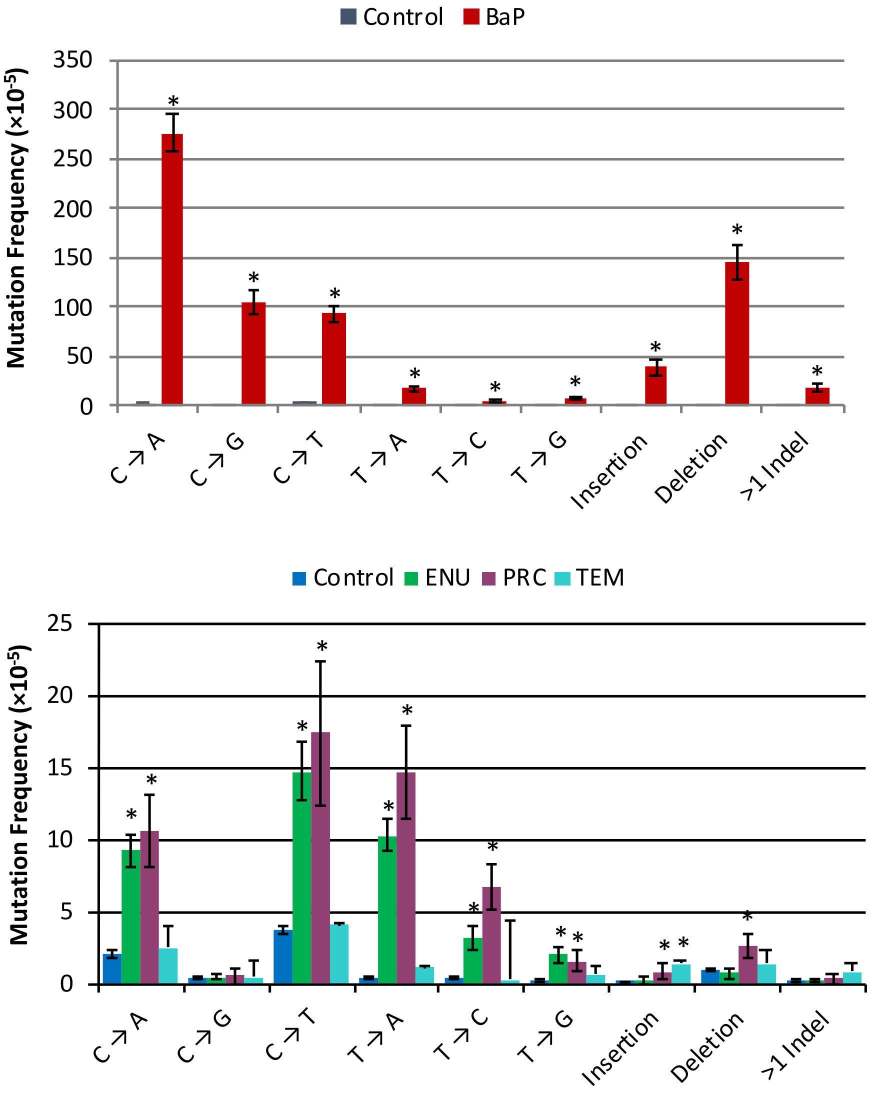

Supplementary Figure 2. Mutation frequencies of each mutation type compared across exposure groups. BaP was presented in its own chart because of its high fold-induction compared to control. Asterisks signifies $P < 0.05$ for mutation type (pairwise comparisons with Bonferroni correction).

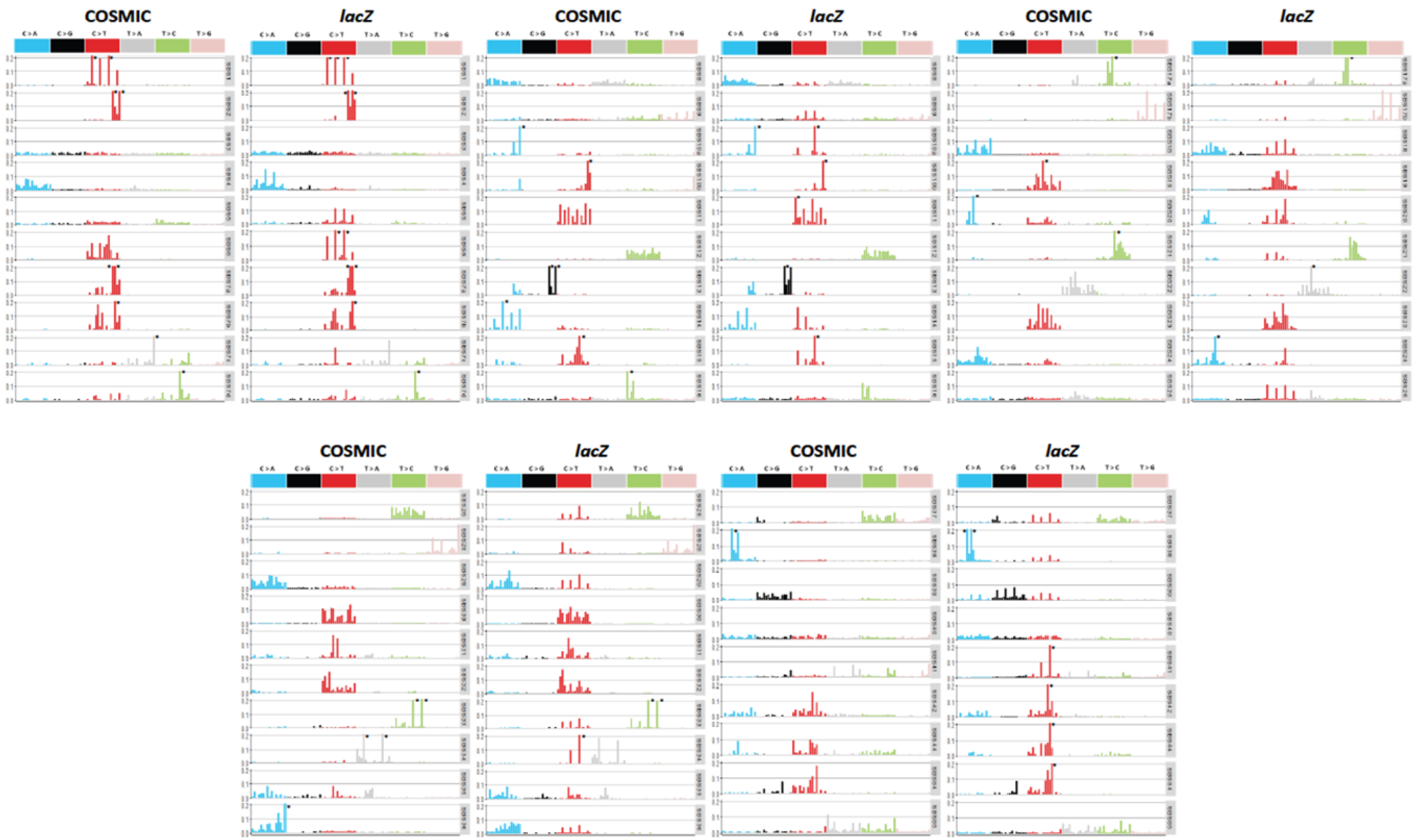

Supplementary Figure 3 - Comparison of COSMIC SBS signatures and *lacZ*-adjusted signatures normalized to the ratio of *lacZ* vs. human trinucleotide frequencies. COSMIC signature data were obtained from the COSMIC database (version 3)

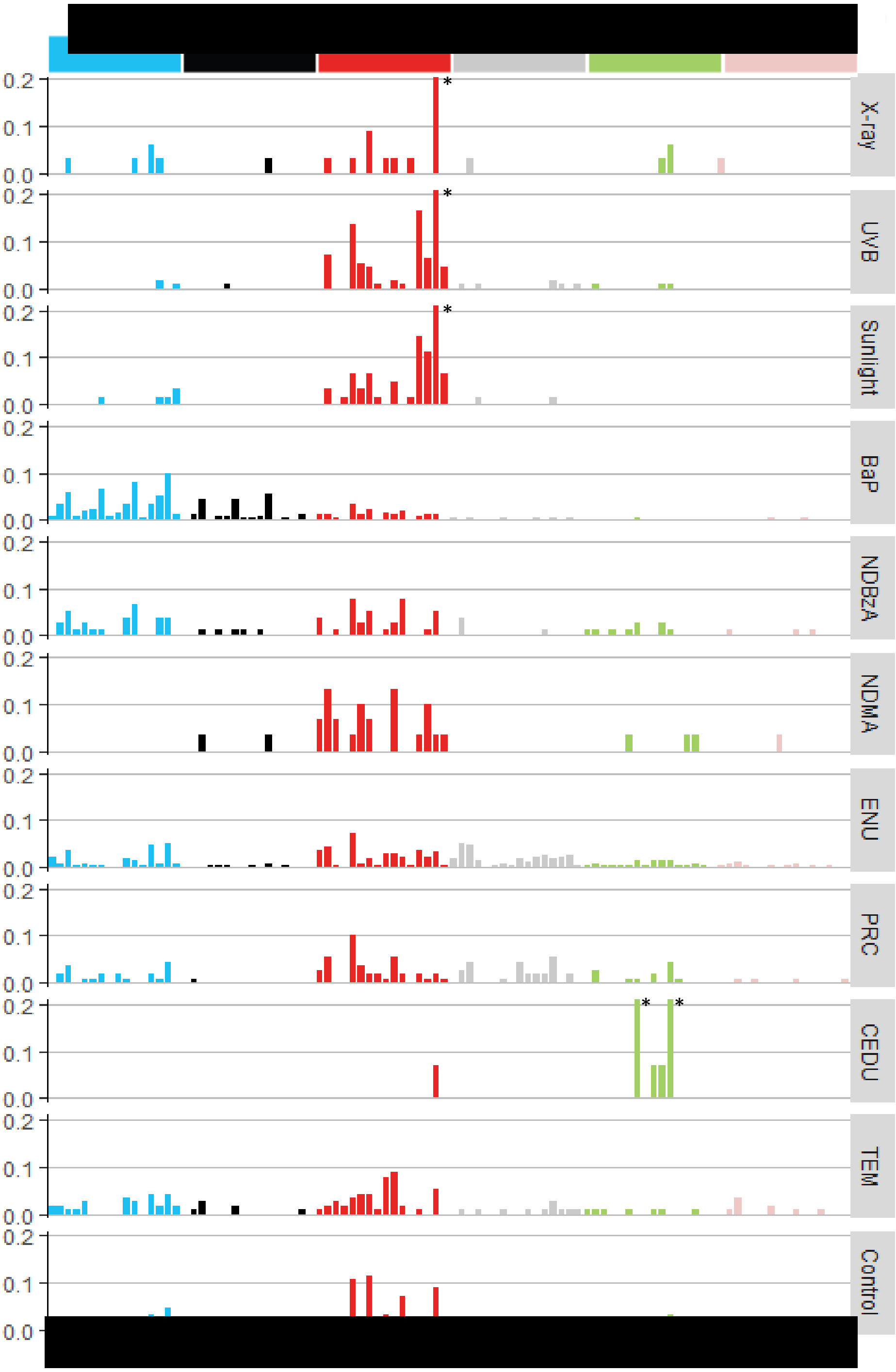

Supplementary Figure 4. The 96-base context mutation spectra catalogued from *in vivo* SNVs characterized by NGS and Sanger from control and chemical-exposed animals.

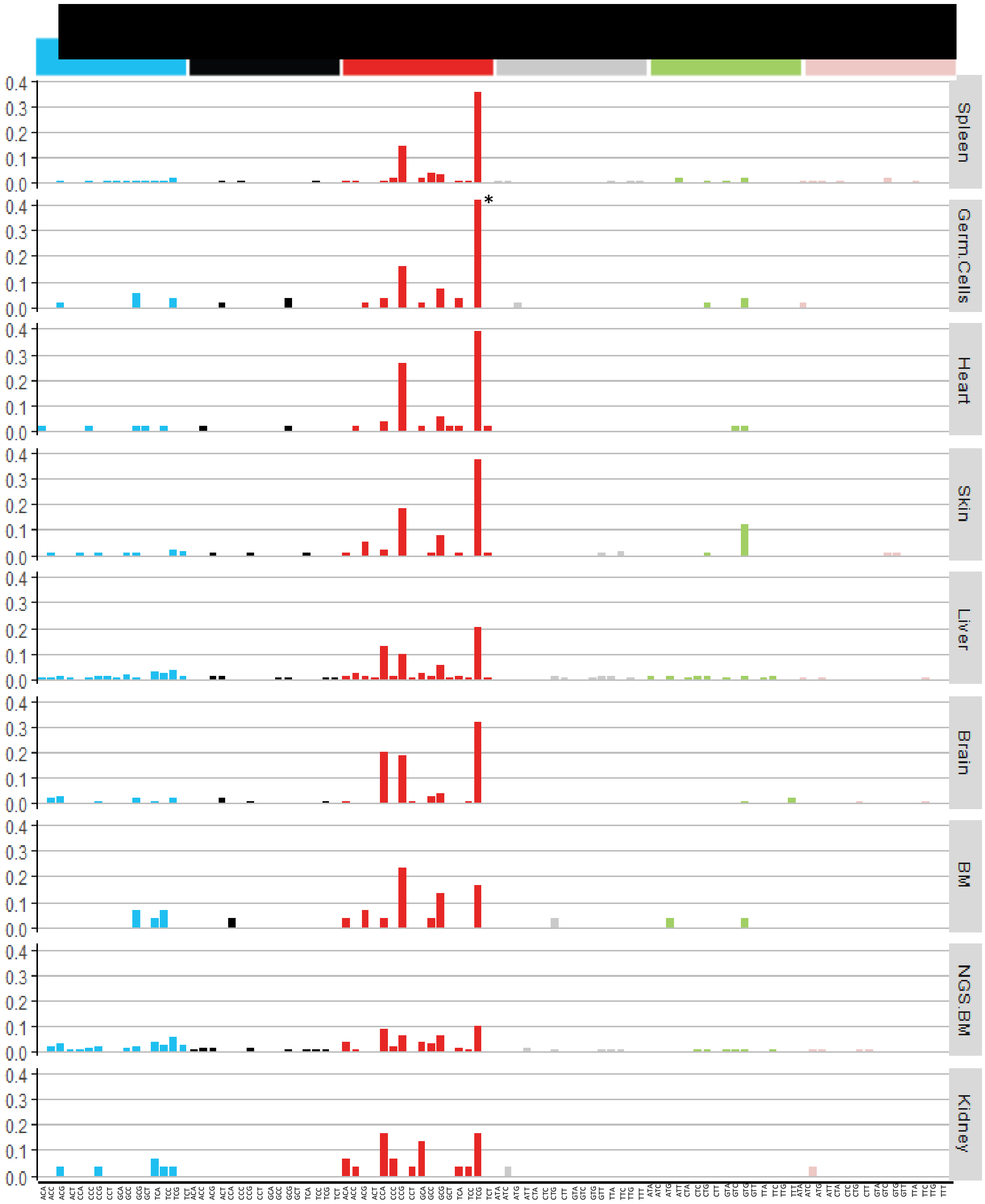

Supplementary Figure 5. The 96-base context mutation spectra catalogued from *in vivo* SNVs characterized in control tissues. Note: the Sanger sequencing results show the SNVs from all positions, including the 5 mutation hotspots. Bone marrow is abbreviated to BM.

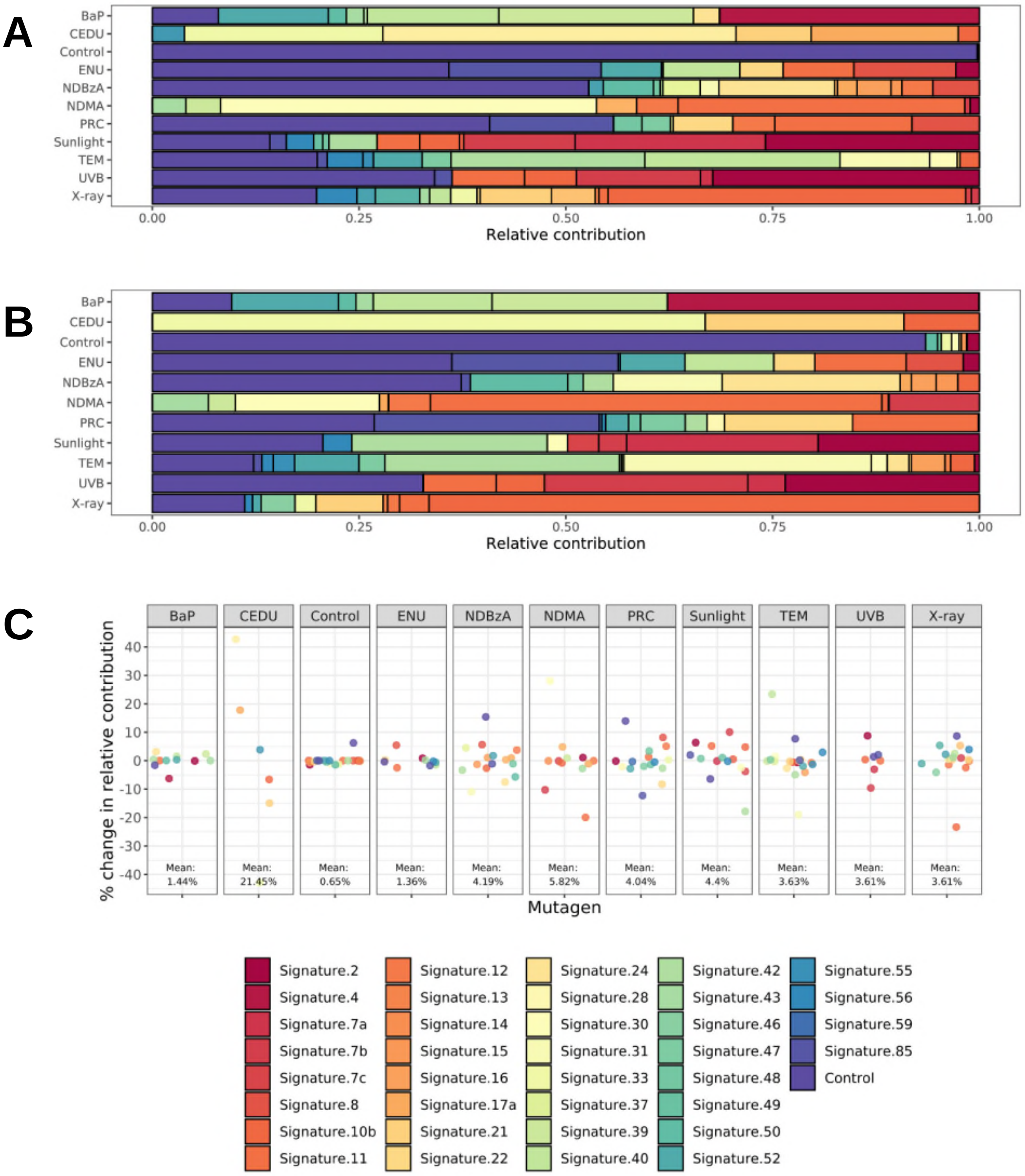

Supplementary Figure 6. Impact of downsampling the number of mutations used to detect COSMIC signatures using MutationalPatterns. The number of mutants used as input into MutationaPatterns was randomly downsampled by 50% to test whether the same COSMIC signatures were detected. (**A**) Results obtained using the complete set of mutations. These

contributions are the same shown in Supplementary Table S4. (**B**) Results obtained with the downsampled data showed that using only 50% of the mutations did not change the signatures were detected. (**C**) Downsampling did change the relative contributions of the detected signatures to some degree. There was a mean of 4.8% absolute difference with respect to when the entire dataset of mutations was used. The absolute difference for each agent is reported at the bottom of corresponding dot plot. The largest difference is observed for CEDU, which was the agent with the smallest number of mutations (n=14).

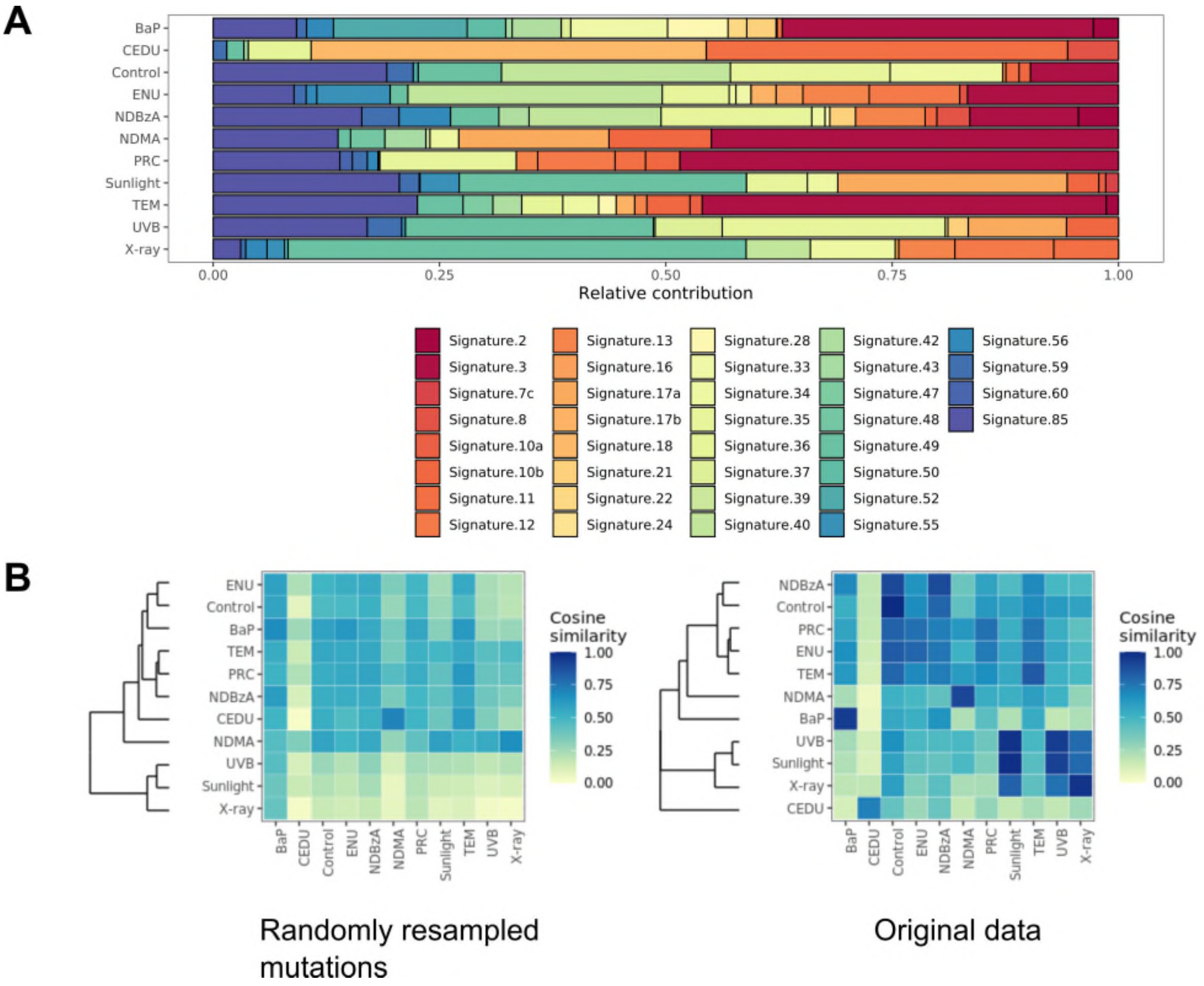

Supplementary Figure 7. **Random resampling of mutations used to query the COSMIC signatures.** The mutation data was resampled by randomly assigning rows in the table without replacement (i.e., the counts for any triplet mutation type could be resampled as any of the other triplet patterns). (**A**) There are no biologically meaningful signatures extracted during resampling, that is, SBS4 is no longer associated with the BaP mutation profile, SBS 2 and SBS 7a are no longer associated with Sunlight and UV, etc. Furthermore, the control signature did not contribute to any of the reconstructions of randomized mutation profiles. (**B**) The reconstructed signatures derived using resampled mutation data have overall lower cosine similarities (left) than the reconstructed signatures using the original mutation data (right). We

conclude that the associations of the detected COSMIC signatures with the mutational patterns of the agents used in this study is not due to chance.

SUPPLEMENTARY TABLES

Supplementary Table 1. Summary of published data from studies that used Sanger sequencing to characterize *lacZ* mutants.

Chemical	Tissues	Total Mutations	SNVs	References[a]
BaP	Colon, Spleen, Stomach	78	60	(38)
CEDU	Bone Marrow	14	14	(41)
ENU	Bone Marrow, Germ Cells, Liver	211	207	(35, 36, 39, 40)
NDBzA	Liver	80	76	(37)
NDMA	Liver	46	30	(34)
Sunlight	Skin	63	62	(30)
UVB	Skin	116	109	(29, 31)
X-Ray	Brain, Liver, Spleen	98	35	(32, 33)

[a]Please see reference section in the manuscript.

Supplementary Table 2. Summary of mutant frequencies measured in control and treated animals.

Group	Number of Animals	Mutants	Plaque Forming Units	Average Mutant Frequency (x10^{-5})	Standard Deviation	Fold Change	P-value
Control[1]	19	403	5,953,396	5.71	2.75	-	-
BaP	6	15,240	2,130,473	701.70	120.93	122.9	**<0.0001**
PRC	8	904	1,597,855	55.13	11.94	9.7	**<0.0001**
ENU	6	446	1,090,523	40.93	9.71	7.2	**<0.0001**
TEM	7	293	2,425,691	9.18	4.53	1.6	**0.048**

[1]The number of control animals and mutants used for mutant frequency calculations are different from the number used for spectral/signature analysis. Mutants from additional control animals were collected for spectral analysis, but plaque forming units were not quantified for these animals.

Supplementary Table 3. Summary of mutants, independent mutations, and unique mutational events characterized in each exposure group by Next Generation Sequencing.

Group	Mutants	Independent Mutations	Unique SNV Events[1]
Control	1046	512	55
BaP	2914	1547	377
PRC	129	120	14
ENU	902	419	85
TEM	428	153	22

[1]There are 3096 positions × 3 possible substitutions for a total of 9288 possible unique SNV events. SNV events were considered unique to a group if they were only observed in that particular group.

Supplementary Table 4. Percent decomposition of SNV data from different agents into COSMIC signatures using deconstructSigs and MutationalPatterns.

Signature	Electromagnetic radiation						Bulky adducts				Alkylating agents						Base analog		Clastogen	
	X rays (34)		UVB (109)		Sunlight (62)		BaP (1165)		NDBzA (76)		NDMA (30)		ENU (611)		PRC (110)		CEDU (14)		TEM (115)	
	DS	MP	DS	MP	DS	MP	DS	MP	DS	MP	DS	MP	DS	MP	DS	MP	DS	MP	DS	MP
2			33	32	27	26						1		3						
4							36	31												
7a			7	1	27	23														
7b		1	15	15	14	13						1								
7c		1																		
8									12	6			16	12	14	8				
10a						1														
10b	49	43	6	6		5												3		2
11		1		9		5					37	35	8	9	17	17				
12										4		5				5				
15										1										
16										4										
17a										2								18		
17b												5								
19		5																		
21	12	9																9		
22														5		7				
24								3	15	14										
26																	80	43		
30											50	45								3
31										2									7	11
33		3																24		
35										5										
39							26	24				1								
40		3					8	16					12	9					33	24
42					7	6													19	23
43		1						2				1								
47																3				3
48						1				1						3				
49						1														
50								2		6										

51	6	5																		
52							15	13					7	7						6
53		2								2										
54																	19	4		
55																				1
56		5				3														1
85				2		2							19	18	19	15				1
Control	25	20	33	34	13	14	9	8	53	53			35	36	42	41			20	20
Residual	8	1	6	1	12	0	6	1	20	0	13	0	3	1	8	1	1	0	21	2
Cosine	0.97	0.97	0.94	0.94	0.98	0.99	0.94	0.95	0.89	0.90	0.90	0.91	0.80	0.81	0.75	0.77	0.67	0.72	0.82	0.85

Number below each agent indicates the number of unique single base substitutions that were sequenced.

DS = deconstructSigs; MP = MutationalPatterns

Supplementary Table 5. Association between SBS signatures and mutation profiles of tested agents with increasing stringency criteria

	SBS signatures contributing to mutation profile				
	Cosine >0.5	Cosine >0.6	Cosine >0.7	Cosine >0.8	Cosine >0.8
X rays	SBS 10b<br>Control	SBS 10b	SBS 10b	SBS 10b	SBS 10b
UVB	SBS 2<br>Control	SBS 2<br>Control	SBS 2	SBS 2	
Sunlight	SBS 2<br>SBS 7a	SBS 2<br>SBS 7a	SBS 2<br>SBS 7a	SBS 2<br>SBS 7a	SBS 7a
BaP	SBS 4<br>SBS 39	SBS 4<br>SBS 39	SBS 4	SBS 4	
NDBzA	Control				
NDMA	SBS 11<br>SBS 30	SBS 11<br>SBS 30	SBS 11<br>SBS 30	SBS 11<br>SBS 30	
ENU	Control	Control			
PRC	Control	Control			
CEDU	SBS 26	SBS 26			
TEM	SBS 40<br>Control	SBS 40<br>Control			

Supplementary Table 6. Percent decomposition of SNV data from different agents into COSMIC signatures (Version 2) using deconstructSigs.

	Electromagnetic Radiation			Bulky Adducts		Alkylating Agents			Base analog	Clastogen
Signature	X rays	UVB	Sunlight	BaP	NDBzA	NDMA	ENU	PRC	CEDU	TEM
2		0.47	0.44							
3				0.25		0.14	0.10			0.23
4	0.14			0.60	0.20					
7	0.32	0.21	0.33							
10	0.49									
11			0.14			0.76		0.12		
12									0.99	
16					0.21		0.46	0.44		0.29
23										0.19
24										
Control		0.28	0.08	0.15	0.49		0.38	0.37		0.28
Residual	0.05	0.04	0.01	0.00	0.10	0.10	0.06	0.07	0.01	0.01
