# Supplementary material for: Chemically induced mutations in a MutaMouse reporter gene inform mechanisms underlying human cancer mutational signatures
Source: Commun Biol. 2020 Aug 14;3:438. doi: 10.1038/s42003-020-01174-y (PMC7429849; doi:10.1038/s42003-020-01174-y)
Supplement: Supplementary file 7 — Description of Additional Supplementary Files [file 42003_2020_1174_MOESM7_ESM.pdf]

Descriptions of Additional Supplementary Files

Supplementary Data 1: Proportions of the various types of mutations by animal. These data were used to generate Figure 2.

Supplementary Data 2: Frequencies of the 96 trinucleotides in the *lacZ* versus the mouse and human genomes. These data were used to generate Figure 3.

Supplementary Data 3: Frequencies of the 96 trinucleotides in the 49 COSMIC Single Base Substitution signatures corrected for the differences in trinucleotide occurrence in the *lacZ* versus the human genome. This dataset was already included in the previous submission and was used to generate Supplementary Figure 3.

Supplementary Data 4: Distribution of sequenced mutations among the 96 possible mutation types for controls and the 10 mutagenic agents. These data were used to generate Figure 5 and Supplementary Figure 4.

Supplementary Data 5: Cosine similarity values between the mutagenic profile of each agent versus the COSMIC SBS signatures. These data were used to generate Figure 4.
