# Supplementary material for: Chemically induced mutations in a MutaMouse reporter gene inform mechanisms underlying human cancer mutational signatures
Source: Commun Biol. 2020 Aug 14;3:438. doi: 10.1038/s42003-020-01174-y (PMC7429849; doi:10.1038/s42003-020-01174-y)
Supplement: Supplementary file 8 — Reporting Summary [file 42003_2020_1174_MOESM8_ESM.pdf]

nature research

Corresponding author(s): Francesco Marchetti

Last updated by author(s): Jul 10, 2020

Reporting Summary

Nature Research wishes to improve the reproducibility of the work that we publish. This form provides structure for consistency and transparency in reporting. For further information on Nature Research policies, see Authors & Referees and the Editorial Policy Checklist.

Statistics

For all statistical analyses, confirm that the following items are present in the figure legend, table legend, main text, or Methods section.

n/a	Confirmed	
☐	☒	The exact sample size (n) for each experimental group/condition, given as a discrete number and unit of measurement
☐	☒	A statement on whether measurements were taken from distinct samples or whether the same sample was measured repeatedly
☐	☒	The statistical test(s) used AND whether they are one- or two-sided<br>*Only common tests should be described solely by name; describe more complex techniques in the Methods section.*
☒	☐	A description of all covariates tested
☐	☒	A description of any assumptions or corrections, such as tests of normality and adjustment for multiple comparisons
☐	☒	A full description of the statistical parameters including central tendency (e.g. means) or other basic estimates (e.g. regression coefficient) AND variation (e.g. standard deviation) or associated estimates of uncertainty (e.g. confidence intervals)
☐	☒	For null hypothesis testing, the test statistic (e.g. F, t, r) with confidence intervals, effect sizes, degrees of freedom and P value noted<br>*Give P values as exact values whenever suitable.*
☒	☐	For Bayesian analysis, information on the choice of priors and Markov chain Monte Carlo settings
☒	☐	For hierarchical and complex designs, identification of the appropriate level for tests and full reporting of outcomes
☐	☒	Estimates of effect sizes (e.g. Cohen's d, Pearson's r), indicating how they were calculated

Our web collection on statistics for biologists contains articles on many of the points above.

Software and code

Policy information about availability of computer code

Data collection	None
Data analysis	The workflow used to do signature analyses are available as an RShiny web-application (https://github.com/MarcBeal/HC-MSD/tree/master/lacZ_Mutations_COSMIC_Signatures and https://github.com/mattjmeier/lacZ_COSMIC; publicly available open source R libraries (eg DecontstructSig)

For manuscripts utilizing custom algorithms or software that are central to the research but not yet described in published literature, software must be made available to editors/reviewers. We strongly encourage code deposition in a community repository (e.g. GitHub). See the Nature Research guidelines for submitting code & software for further information.

Data

Policy information about availability of data

All manuscripts must include a data availability statement. This statement should provide the following information, where applicable:

- Accession codes, unique identifiers, or web links for publicly available datasets
- A list of figures that have associated raw data
- A description of any restrictions on data availability

Sequenced mutations generated in-house for BaP, ENU, PRC and TEM are available on the Sequence Read Archive under BioProject accession number PRIJNA 640660. Sequenced mutants from all other agents were obtained from the published literature. Sequenced mutants from all other agents were obtained from the published literature.

Field-specific reporting

Please select the one below that is the best fit for your research. If you are not sure, read the appropriate sections before making your selection.

☒ Life sciences ☐ Behavioural & social sciences ☐ Ecological, evolutionary & environmental sciences

For a reference copy of the document with all sections, see nature.com/documents/nr-reporting-summary-flat.pdf

Life sciences study design

All studies must disclose on these points even when the disclosure is negative.

Sample size	Number of mutations are reported for all agents presented in the study
Data exclusions	None
Replication	No
Randomization	No
Blinding	No, but all analyses were conducted by automated pipelines.

Reporting for specific materials, systems and methods

We require information from authors about some types of materials, experimental systems and methods used in many studies. Here, indicate whether each material, system or method listed is relevant to your study. If you are not sure if a list item applies to your research, read the appropriate section before selecting a response.

Materials & experimental systems

n/a	Involved in the study
☒	☐ Antibodies
☒	☐ Eukaryotic cell lines
☒	☐ Palaeontology
☐	☒ Animals and other organisms
☒	☐ Human research participants
☒	☐ Clinical data

Methods

n/a	Involved in the study
☒	☐ ChIP-seq
☒	☐ Flow cytometry
☒	☐ MRI-based neuroimaging

Animals and other organisms

Policy information about studies involving animals; ARRIVE guidelines recommended for reporting animal research

Laboratory animals	MutaMouse males produced from an in-house colony that has been maintained at Health Canada for over 25 years
Wild animals	Not applicable
Field-collected samples	Not applicable
Ethics oversight	All animal handling, exposures and procedures were conducted according to conditions approved by the Health Canada Ottawa Animal Care Committee before the beginning of the studies

Note that full information on the approval of the study protocol must also be provided in the manuscript.
